# Glycaemic control for people with type 2 diabetes in Saudi Arabia – an urgent need for a review of management plan

**DOI:** 10.1186/s12902-018-0292-9

**Published:** 2018-09-10

**Authors:** Mohammed J. Alramadan, Dianna J. Magliano, Turky H. Almigbal, Mohammed Ali Batais, Afsana Afroz, Hesham J. Alramadhan, Waad Faozi Mahfoud, Adel Mehmas Alragas, Baki Billah

**Affiliations:** 1 Department of Epidemiology and Preventive Medicine, School of Public Health and Preventive Medicine, Monash University, Melbourne, Australia; 2 Baker Heart and Diabetes Institute, Melbourne, VIC Australia; 3 College of Medicine, King Saud University, Riyadh, Saudi Arabia; 4 College of Medicine, King Faisal University, Al-Ahsa, Saudi Arabia; 5 Ibn Sina National College, Jeddah, Saudi Arabia; 6 Medical City - King Saud University, Riyadh, Saudi Arabia

**Keywords:** Saudi Arabia, Diabetes, Glycaemic control

## Abstract

**Background:**

The aim of this study was to assess inadequate glycaemic control and its associated factors among people with type 2 diabetes in Saudi Arabia.

**Methods:**

A cross-sectional study design was used. Adults with type 2 diabetes attending diabetes centres in Riyadh, Hofuf and Jeddah cities were interviewed and their anthropometrics were measured. Their medical records were also reviewed to collect information related to recent lab tests, medications, and documented comorbidities. Multivariable logistic regression were used for data analysis.

**Results:**

A total of 1111 participants were recruited in the study. Mean age was 57.6 (±11.1) years, 65.2% of the participants were females, and mean HbA1c was 8.5 ± 1.9%. About three-fourths of participants had inadequate glycaemic control (≥ 7%). Multivariable analysis showed that age ≤ 60 years, longer duration of diabetes, living in a remote location, low household income, low intake of fruits and vegetable, low level of physical activity, lack of knowledge about haemoglobin A1c, high waist-hip ratio, low adherence to medication, and using injectable medications were independent risk factors for inadequate glycaemic control.

**Conclusions:**

Inadequate glycaemic control is prevalent among people with type 2 diabetes in Saudi Arabia. In order to improve glycaemic control diabetes management plan should aim at controlling the modifiable risk factors which include low intake of fruits and vegetable, low level of physical activity, lack of knowledge about haemoglobin A1c, high waist-hip ratio, and low adherence to medications.

## Background

Diabetes mellitus is a major worldwide public health issue. A recent study showed that the prevalence of diabetes mellitus in Saudi Arabia was 13.4% [1], which is higher than the global prevalence of 8.8% and the prevalence in the Middle East of 10.7% [2]. In fact, Saudi Arabia is among the countries with the highest prevalence of diabetes regionally and globally [2], and the prevalence of diabetes in the country is on the rise [3].

The primary goal of the management of diabetes is to maintain blood glucose levels within or close to normal ranges [4]. It is well established that elevated blood glucose level increases the risk of diabetes complications and mortality [5, 6], while intensive glycaemic control substantially lower the risk [7, 8]. Unfortunately, studies from Saudi Arabia showed that half to two-thirds of people with type 2 diabetes mellitus (T2DM) have poor glycaemic control [9–13], and the prevalence of diabetes complications is higher than the global and regional prevalence [14–16].

A few studies have examined risk factors of poor glycaemic control among people with T2DM in Saudi Arabia [9, 10, 12, 13, 17]. Many of these studies were limited to a small geographical location and included a relatively small cohort of participants. In addition, some of these studies used random or fasting blood sugar test to assess glycaemic control which is not as accurate as the glycated haemoglobin A1c (HbA1c) level that measures the control over a number of weeks [4]. Moreover, not all potential risk factors of poor control were explored in the previous studies. A systematic review and meta-analysis showed that among other factors, diet and physical activity were significantly associated with glycaemic control [18]. Studies from Saudi Arabia, however, did not adequately explore the effect of physical activity and eating habits.

To achieve glycaemic control goals among people with T2DM, all possible associated risk factors of inadequate control must be identified and considered in the management. The aim of this study was to assess the current status of glycaemic control and to identify risk factors for inadequate control among people with T2DM in Saudi Arabia.

## Methods

This study was conducted as a part of a research project that examined the status of glycaemic control, diabetes complications and quality of life for people with T2DM in Saudi Arabia. The study methodology has been described in detail in another article [19]. Ethical approval was obtained from the Monash University Human Research Ethics Committee in Australia and the Research Ethics Committee of the Ministry of Health in Saudi Arabia. All the study procedures were carried out in accordance with the principles of the Declaration of Helsinki as revised in 2013.

### Participants

The study population were people with T2DM aged 18 years and over who were followed up at diabetes centres in three major cities (Hofuf, Riyadh, and Jeddah) in Saudi Arabia. The plan was to recruit 1082 participants based on a sample size calculation with 90% power, 5% significance level, a margin of error of 2.5%, and a prevalence of poor glycaemic control of 50% [9, 20]. Inclusion criteria include documented diagnosis of T2DM, aged 18 years and above, and duration of diabetes of at least 1 year. Pregnant women and participants who did not have a HbA1c test within 1 year were excluded.

### Data collection

Participants were selected randomly from consecutive attendees of the diabetic centres between May 15 and November 30, 2017. After explaining the study and obtaining informed consent in writing, participants were interviewed using a pre-tested structured electronic questionnaire through Research Electronic Data Capture (REDCap) application [19, 21]. The questionnaire collects information related to socio-demographics, lifestyle, medical history, cognitive function, anxiety and depression. Socio-demographic data include gender, date of birth, marital status, education, and income. Lifestyle data include smoking status, physical activity (Global Physical Activity Questionnaire (GPAQ) [22]), and dietary habits. The dietary habit section included 10 questions selected from the UK diabetes and diet questionnaire (UKDDQ) [23] with some modifications to make some points more suitable for the eating habits of the Saudi population. The dietary questions include the frequency of consuming fruits, vegetables, red meat, desserts, date, sugary drinks, butter, bread, and rice and the fat content of consumed milk. The dietary habit variable was measured as a scale between zero and 48, where high score means the individual is following healthy eating habits. Medical history data include the duration of diabetes, modality of treatment, follow-up centre, other comorbidities, medication adherence (the 4-item Morisky Medication Adherence questionnaire) [24], family support in regards to diet and physical activity, and peripheral neuropathy (the Michigan Neuropathy Screening Instrument) [25]. Mental status data include anxiety (Generalized Anxiety Disorder Scale (GAD-2)) [26], depression (the Patient Health Questionnaire-2 (PHQ-2)) [27], and cognitive function (Rowland Universal Dementia Assessment Scale (RUDAS)) [28]. The establishers’ permission to use the above-mentioned tools was obtained.

After interviewing participants, their anthropometrics were measured and recorded. Blood pressure was measured three times after sitting for at least 10 min using the Omron BP742N5 Series Upper Arm Blood Pressure Monitor with a cuff that fits standard and large arms. Weight was measured after instructing participants to remove their shoes and outer layers of clothing. Height, waist circumference, and hip circumference were measured twice and if the measurement varied by more than 2 cm, a third measurement was taken. Waist and hip circumference were measured against thin clothing. Participants’ medical records were reviewed for most recent lab test results, namely HbA1c, creatinine, cholesterol, high density lipoprotein (HDL), low density lipoprotein (LDL), and triglycerides. Information regarding currently prescribed medication, and documented diagnosis of hypertension, coronary artery disease, retinopathy, and stroke was also collected from medical records.

### Operational definitions

Glycaemic control was categorised into controlled (HbA1c < 53 mmol/mol (< 7%)), partially controlled (HbA1c 53–63 mmol/mol (7% to < 8%)), and poorly controlled (HbA1C ≥ 64 mmol/mol (≥ 8%)) [4]. A HbA1c cut-off value of > 68 mmol/mol (≥ 9%) was also used to represent very poor control [29]. Using the Global Physical Activity Questionnaire (GPAQ) [22], the total number of minutes of physical activity per week was categorised into ≥150 min and < 150 min [4]. Treatment modalities was categorized as oral tablets only, injectable medications (insulin and glucagon-like peptide-1 receptor agonists) only, and combined (oral tablets and injectable medications). Using the Morisky Medication Adherence questionnaire [24] medication adherence was categorized into adequate (a score of zero), and inadequate (a score of one or more). Hypoglycaemia was defined as episodes of mild or severe hypoglycaemia symptoms including hunger sweating, light headedness, headache, shaking, trembling, weakness, dizziness, confusion, loss of consciousness, and seizures. Family support in regards to healthy diet and physical activity was categorised as good support if healthy diet and physical activity were encouraged all the time, inadequate support if encouragement was sometimes, and poor support if family members rarely or never encouraged healthy diet or physical activity. Participants were considered unaware of HbA1c if they had not heard of HbA1c before or they did not know the recommended HbA1c target for people with diabetes. Body Mass Index (BMI) was categorised according to the current World Health Organization guidelines into normal (< 25.0 kg/m^2^), pre-obesity (25.0–29.9 kg/m^2^), and obesity (class I, II and II ≥ 30.0 kg/m^2^). High waist-hip ratio was defined as a ratio of > 0.96 for men and >  0.98 for women [30]. Hypertension was defined as either a documented diagnosis of hypertension, taking antihypertension medications, or three previous high blood pressure readings (systolic ≥140 or diastolic ≥90) [4]. Dyslipidaemia was defined as any of the following: total cholesterol > 4.0 mmol/L (154.7 mg/dl), LDL > 2.0 mmol/L (77.3 mg/dl), triglycerides > 2.0 mmol/L (177.1 mg/dl), or HDL < 1.0 mmol/L (38.7 mg/dl) [31]. Impaired cognitive function was defined as a score of ≤22 in the Rowland Universal Dementia Assessment Scale (RUDAS) [28]. Depression was defined as a score of three and more using the Patient Health Questionnaire-2 (PHQ-2)) [27]. Anxiety was defined as a score of three and more using the Generalized Anxiety Disorder Scale (GAD-2)) [26]. Macrovascular complications were defined as having one of the following: documented diagnosis of stroke (irreversible cerebrovascular accident), documented diagnosis of coronary artery disease, taking medication for coronary artery disease, underwent a procedure for coronary artery disease, or self-reported lower extremity ulcers or amputations. Microvascular complication was defined as having one of the following: documented diagnosis of retinopathy, the participant had been told by an ophthalmologist that he or she had retinopathy, a score of seven or more using the Michigan Neuropathy Screening Instrument [25], or estimated glomerular filtration rate ≤ 60 ml/min/1.73m^2^ calculated from serum creatinine using the Chronic Kidney Disease Epidemiology Collaboration (CKD-EPI) equation [32, 33].

### Data analysis

Stata SE version 15.0 was used for data analysis. Data were summarised and presented as a mean (± standard deviation) for numerical data and frequency and percentage for categorical data. ANOVA and chi-square tests were used to examine univariate associations between risk factors and levels of glycaemic control. Potential risk factors with a *p*-value of 0.2 from univariate analysis were entered into multivariable logistic regression with step wise variable selection [34]. In the regression analysis, glycaemic control was categorised into controlled (HbA1c < 53 mmol/mol (< 7%)) and inadequately controlled (HbA1c ≥ 53 mmol/mol (≥ 7%)). The determinants were also examined for very poor glycaemic control (HbA1c > 68 mmol/mol (≥ 9%)). A *p*-value of 0.05 or less was considered as statistical significant.

## Results

A total of 1111 participants were recruited in this study; 624 participants (56.2%) were from Riyadh, 239 (21.5%) were from Hofuf, and 248 (22.3%) were from Jeddah. Four hundred and-fifty participants (40.5%) were followed up at diabetes centres only, while 125 participants (11.3%) were followed up at both diabetes centres and hospitals and 535 participants (48.2%) were followed up at both diabetes centres and primary healthcare centres. Mean age was 57.6 (±11.1) years, mean duration of diabetes was 13.9 (±8.4) years, and 65.2% (724) of the participants were females, while 34.8% (387) were males. Mean body mass index was 32.9 (±8.1) kg/m^2^. Mean HbA1c was 69.4 (±15.5) mmol/mol (8.5% (±1.9%)), and 24.1% of participants had good glycaemic control (HbA1c < 53 mmol/mol (< 7%)), 21.7% had partial control (HbA1c 53–63 mmol/mol (7–7.9%)), and 54.2% had poor control (HbA1C ≥ 64 mmol/mol (≥ 8%)). None of the study participants were on insulin pump or continuous glucose monitoring.

Table 1 summarises participants’ demographic and lifestyle characteristics by different levels of glycaemic control. There was a higher prevalence of poor glycaemic control among those with lower levels of education (*p*-value: < 0.001), living in a remote place (*p*-value: 0.002), and not working including house-wives (*p*-value: 0.005). A higher proportion of those aged 60 years and younger had poor glycaemic control, however, the association was not statistically significant in univariate analysis. Similarly, gender, nationality, household income, and region were not significantly related to glycaemic control. Regarding lifestyle factors, there was no difference in the mean of healthy eating habit score among different categories of glycaemic control. However, higher proportions of those with less than daily intake of fruits and vegetables had poor glycaemic control (*p*-value: 0.011). Similarly, higher proportions of those with physical activity less than 150 min per week had poor control (*p*-value: 0.032). Mean number of sitting hours and smoking did not differ significantly among the categories of glycaemic control.

**Table 1:** Demographic and lifestyle characteristics by level of glycaemic control

Variable	Glycaemic control			*P*-value
	Good (HbA1c < 7.0%) *n* = 263	Partial (HbA1c 7.0% - 7.9) *n* = 237	Poor (HbA1c ≥ 8%) *n* = 592	
Age % (n)				
> 60 years	28.4 (109)	22.4 (86)	49.2 (189)	0.106
46–60 years	21.4 (123)	21.6 (124)	57.0 (327)	
< 46 years	23.1 (31)	20.2 (27)	56.7 (76)	
Gender: female % (n)				
Female	22.9 (162)	20.9 (148)	56.3 (399)	0.173
Male	26.4 (101)	23.2 (89)	50.4 (193)	
Nationality % (n)				
Saudi	23.6 (246)	21.7 (226)	54.7 (570)	0.207
Non-Saudi	34.0 (17)	22.0 (11)	44.0 (22)	
Education level % (n)				
University/college	30.9 (60)	28.9 (56)	40.2 (78)	< 0.001
Lower education level	22.6 (203)	20.2 (181)	57.2 (514)	
Location of residency % (n)				
Urban	24.3 (229)	22.9 (216)	52.8 (497)	0.002
Rural	30.4 (28)	15.2 (14)	54.4 (50)	
Remote	10.3 (6)	12.1 (7)	77.6 (45)	
Working status % (n)				
Working	23.9 (55)	20.4 (47)	55.7 (128)	0.005
Not working / house-wife	21.6 (141)	21.0 (137)	57.4 (374)	
Retired	31.9 (67)	25.2 (53)	42.9 (90)	
Household income % (n)				
≥ 6001 SAR	25.2 (148)	23.7 (139)	51.1 (300)	0.074
< 6001 SAR	22.8 (115)	19.4 (98)	57.8 (292)	
Region % (n)				
Riyadh	26.4 (161)	22.5 (137)	51.1 (311)	0.125
Jeddah	22.7 (56)	21.5 (53)	55.9 (138)	
Hofuf	19.5 (46)	19.9 (47)	60.6 (143)	
Active smoking % (n)				
Never	24.6 (230)	21.9 (205)	53.5 (501)	0.563
In the past (> 1 year)	20.7 (19)	17.4 (16)	62.0 (57)	
Current smoker	22.2 (14)	25.4 (16)	52.4 (33)	
Eating habit score (mean ± SD)	30.2 ± 4.4	29.6 ± 5.1	29.8 ± 5.1	0.451
Fruits and vegetables % (n)				
Daily	31.3 (76)	20.2 (49)	48.6 (118)	0.011
Less frequent	21.9 (186)	22.2 (188)	55.9 (474)	
Physical Activity % (n)				
≥ 150 min/week	29.0 (94)	21.9 (71)	49.1 (159)	0.032
< 150 min/week	22.0 (169)	21.6 (166)	56.4 (433)	
Sitting hours (mean ± SD)	6.1 ± 3.6	5.8 ± 3.5	6.2 ± 3.8	0.380

The association between various clinical characteristics and glycaemic control are presented in Table 2. Higher proportions of poor control were among people with longer duration of diabetes (*p*-value: < 0.001), and who were taking injectable medications (*p*-value: < 0.001), followed up mainly at primary health care or diabetes centres (*p*-value: 0.019), used glucometer twice or more a week (*p*-value: 0.017), and were not aware of HbA1c or the recommended HbA1c target for people with diabetes (*p*-value: 0.016). Similarly, higher proportions of poor control were among those who had less frequent hypoglycaemia events (*p*-value: 0.016), macrovascular complications (*p*-value: 0.019), microvascular complications (*p*-value < 0.001), and high waist-hip ratio (*p*-value: 0.001). Other clinical characteristics including family history of diabetes, family support, hypertension, adherence to medication, depression, anxiety, cognitive function, dyslipidaemia, and body mass index, did not appear to have an association with glycaemic control.

**Table 2:** Clinical characteristics by level of glycaemic control

Variable	Glycaemic control			*P*-value
	Good (HbA1c < 7.0%) *n* = 263	Partial (HbA1c 7.0% - 7.9) *n* = 237	Poor (HbA1c ≥ 8%) *n* = 592	
Diabetes Duration % (n)				
≤ 10 years	34.7 (143)	25.5 (105)	39.8 (164)	< 0.001
> 10 years	17.5 (119)	19.4 (132)	63.0 (428)	
Family history of diabetes % (n)				
Yes	22.9 (187)	22.2 (181)	54.9 (447)	0.325
No	27.2 (75)	20.3 (56)	52.5 (145)	
Modality of treatment				
Oral tablets	36.4 (219)	25.8 (155)	37.9 (228)	< 0.001
Injectable	9.9 (22)	18.5 (41)	71.6 (159)	
Oral and injectable	7.9 (21)	15.4 (41)	76.8 (205)	
Main follow up centre % (n)				
Hospital	34.2 (42)	22.0 (27)	43.9 (54)	0.019
Primary care centre	24 (125)	23.0 (120)	53.0 (276)	
Diabetes centre	21.3 (95)	20.1 (90)	58.6 (262)	
Glucometer use % (n)				
Once or more a week	21.4 (145)	21.7 (147)	57.0 (387)	0.017
Less than once a week	28.6 (118)	21.8 (90)	49.6 (205)	
Hypoglycaemia events % (n)				
None	26.9 (170)	21.1 (133)	52.0 (328)	0.016
1–5 times	18.2 (71)	22.8 (89)	59.1 (231)	
6 times and more	30.4 (21)	21.7 (15)	47.8 (33)	
Medication adherence % (n)				
Adequate	26.0 (165)	22.4 (142)	51.7 (328)	0.110
Inadequate	21.4 (98)	20.8 (95)	57.8 (264)	
Family support with diet % (n)				
Good	23.5 (103)	22.2 (97)	54.3 (238)	0.621
Inadequate	23.0 (73)	23.9 (76)	53.1 (169)	
Poor	25.9 (87)	19.1 (64)	55.1 (185)	
Family support with physical activity % (n)				
Good	21.4 (66)	21.0 (65)	57.6 (178)	0.460
Inadequate	23.2 (74)	23.2 (74)	53.6 (171)	
Poor	26.5 (123)	21.1 (98)	52.4 (243)	
knowledge about HbA1c % (n)				
Aware	29.5 (103)	20.3 (71)	50.1 (175)	0.016
Not aware	21.5 (160)	22.3 (166)	56.1 (417)	
Body mass index % (n)				
Underweight/normal	22.7 (25)	20.9 (23)	56.4 (62)	0.075
Pre-obesity	26.4 (78)	26.4 (78)	47.1 (139)	
Obesity (class I – III)	23.3 (158)	20.0 (136)	56.7 (385)	
Waist-hip ratio % (n)				
Normal	26.4 (144)	24.8 (135)	48.8 (266)	0.001
High (male: > 0.96, female: > 0.98)	22.1 (96)	17.5 (76)	60.5 (263)	
Depression % (n)				
No	24.1 (220)	22.3 (203)	53.6 (489)	0.563
Yes	23.9 (43)	18.9 (34)	57.2 (103)	
Anxiety % (n)				
No	24.2 (224)	22.4 (207)	53.4 (493)	0.328
Yes	23.2 (39)	17.9 (30)	58.9 (99)	
Cognitive function % (n)				
Intact	24.3 (169)	21.4 (149)	54.2 (377)	0.709
Impaired	22.1 (59)	23.2 (62)	54.7 (146)	
Dyslipidaemia % (n)				
No	29.6 (45)	23.0 (35)	47.4 (72)	0.142
Yes	23.2 (218)	21.5 (202)	55.3 (520)	
Hypertension % (n)				
No	22.8 (74)	20.3 (66)	56.9 (185)	0.503
Yes	24.6 (189)	22.3 (171)	53.1 (407)	
Macrovascular complications				
No	25.3 (199)	23.1 (182)	51.6 (406)	0.019
Yes	21.0 (64)	18.0 (55)	61.0 (186)	
Microvascular complication				
No	29.4 (146)	23.7 (118)	46.9 (233)	< 0.001
Yes	19.7 (117)	20.0 (119)	60.3 (359)	

**Fig. 1:**
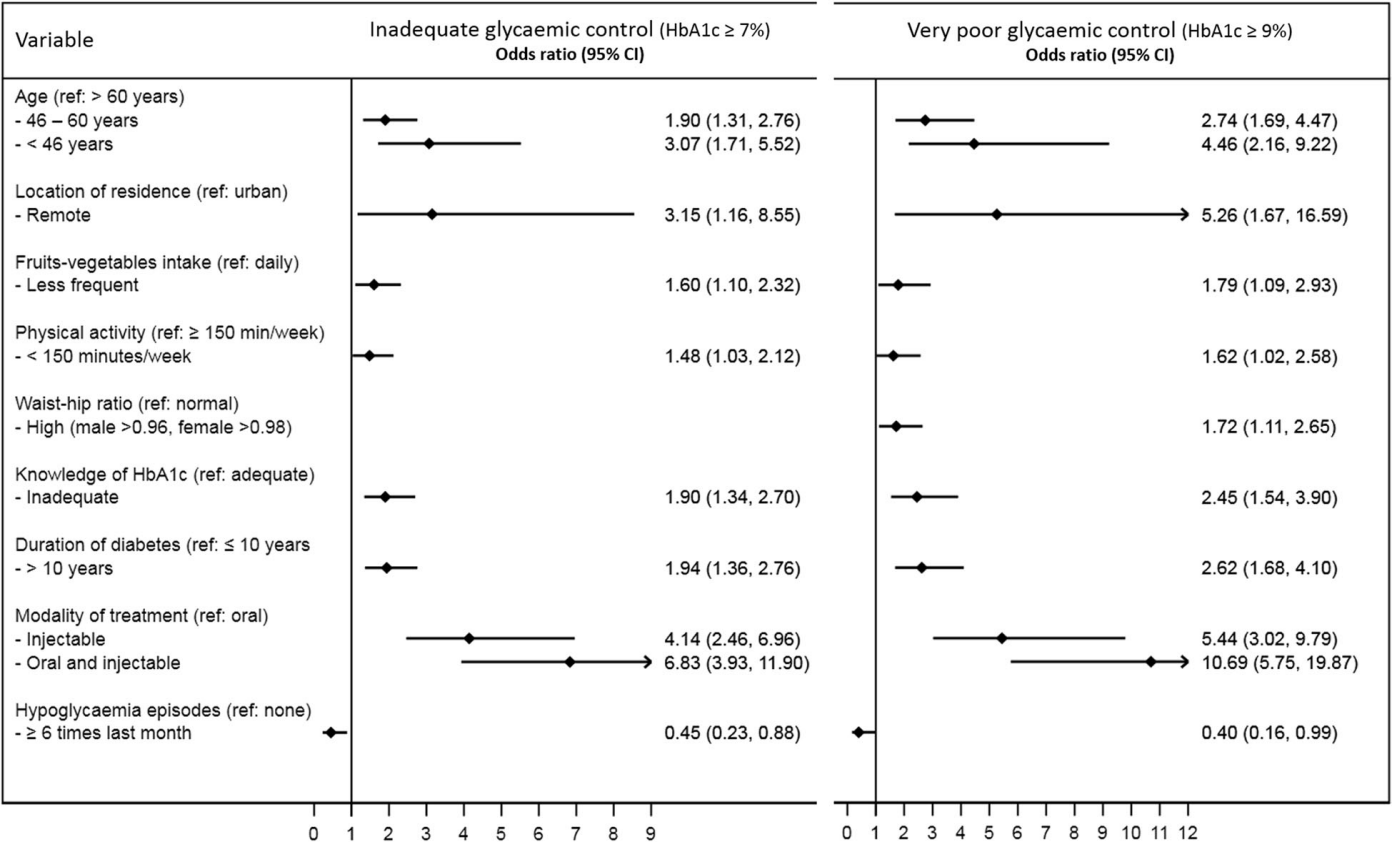
Adjusted association between risk factors and inadequate (HbA1c ≥ 7%) and very poor (HbA1c ≥ 9%) glycaemic control. Variables introduced in the multivariable analysis were age, gender, education level, location of residence, work status, income, region, intake of fruits and vegetables, physical activity, duration of diabetes, treatment modality, glucometer use frequency, hypoglycaemia, follow-up location, adherence to medication, awareness of HbA1c, BMI, waist-hip ratio, macrovascular complications, microvascular complications and dyslipidaemia

Figure 1 summarises the results of the multivariable logistic regression analysis. A total of 379 participants (34.1%) had very poor glycaemic control (HbA1c > 68 mmol/mol (> 9%)). Less than daily intake of fruits and vegetables increased the risk of inadequate and very poor control by 60% and 79% respectively. Low level of physical activity was associated with 48% and 62% higher risk of inadequate and very poor control respectively. Inadequate knowledge of HbA1c was associated with 1.9-fold and 2.5-fold higher risk of inadequate and very poor control respectively. High waist-hip ratio increased the risk of very poor control by 72%, while frequent episodes of hypoglycaemia is associated with lower risk of both inadequate and very poor control. Other risk factors that were associated with inadequate and very poor control include younger age, longer duration of diabetes, remote location of residence, and using injectable medications with or without oral tablets.

**Fig. 2:**
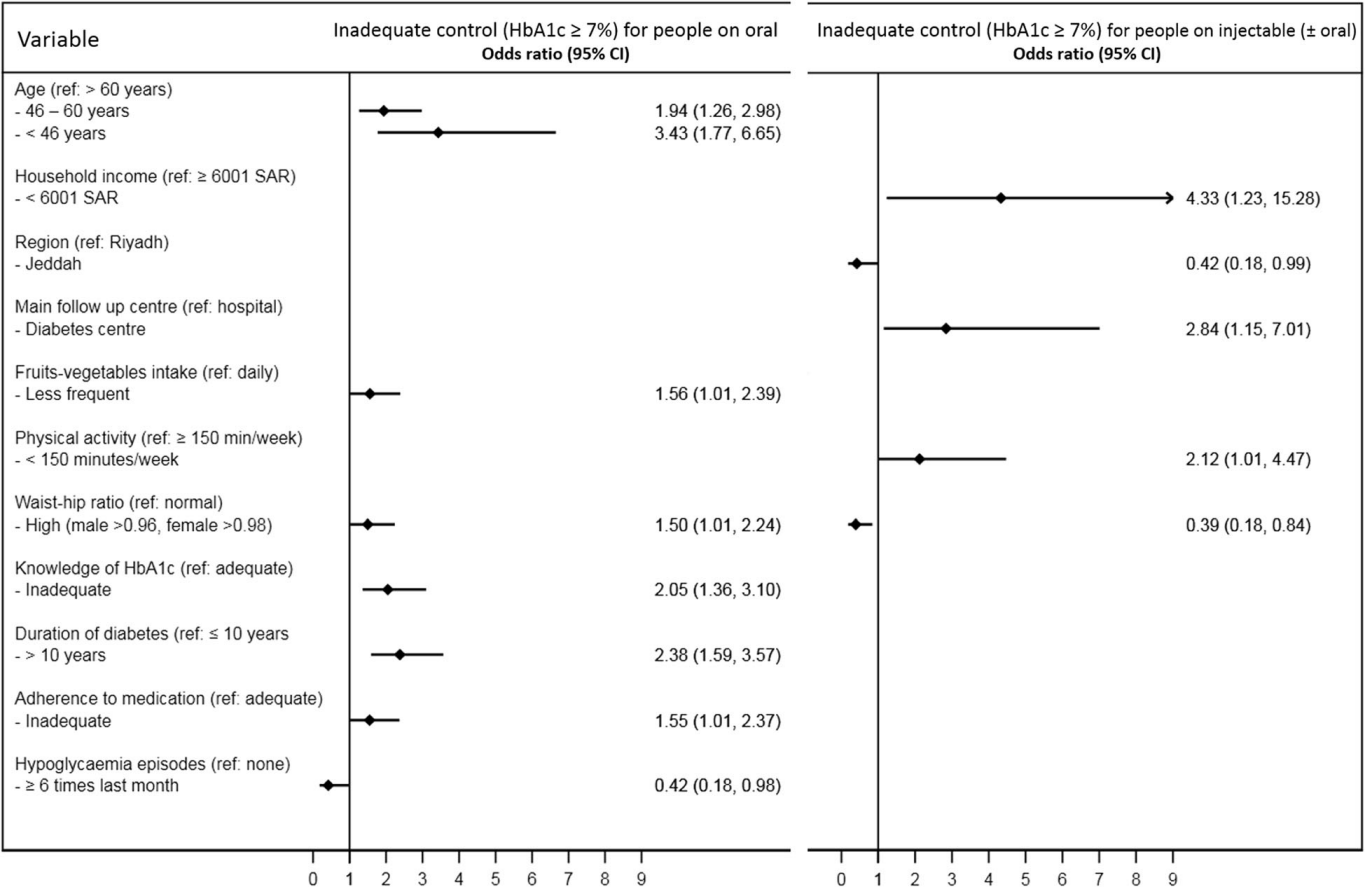
Adjusted association between risk factors and inadequate control (HbA1c ≥ 7%) by the modality of treatment. Variables introduced in the multivariable analysis were age, gender, education level, location of residence, work status, income, region, intake of fruits and vegetables, physical activity, duration of diabetes, treatment modality, glucometer use frequency, hypoglycaemia, follow-up location, adherence to medication, awareness of HbA1c, BMI, waist-hip ratio, macrovascular complications, microvascular complications and dyslipidaemia

Figure 2 illustrates the adjusted association between risk factors and inadequate control (HbA1c ≥ 7%) for people who were on oral tablets only as well as for those on injectable medications (with or without oral tablets). Among people on oral tablets the risk of inadequate control was higher by: 56% for low intake of fruits and vegetables, 50% for high waist-hip ratio, and by 55% for inadequate adherence to medication. Inadequate knowledge of HbA1c was associated with 2.1-fold increased risk among those on oral tablets, while frequent hypoglycaemia reduced the risk by 58%. Other risk factors of inadequate control among those on oral tablets include younger age and longer diabetes duration. For participants who were on injectable medications, low level of physical activity increased the risk of inadequate control by 2.1-folds, while high waist-hip ratio reduced the risk by 61%. Other risk factors of inadequate control among those on injectable medications were lower household income and followed up mainly at diabetes centres, while people from Jeddah appeared to have lower risk.

## Discussion

In this current multi-centre study we have assessed the status of glycaemic control and its associated factors among people with T2DM attending diabetes centres in Saudi Arabia. One important finding was that only 24.1% of people with T2DM achieved the recommended HbA1c level of less than < 53 mmol/mol (< 7%), while the majority (75.9%) did not attained this target. Our findings, however, were comparable to the findings of recent studies from Saudi Arabia and other Arabian Gulf Countries [10–13, 35–39]. Despite quality health care services and various antidiabetic medications that are available for people with diabetes at no cost, the majority of people with T2DM in Saudi Arabia continue to have inadequate glycaemic control. A possible explanation could be the embracing of unhealthy lifestyle.

Sedentary lifestyle and the consumptions of processed, energy condensed, and fat-rich food have led to the increased prevalence of obesity and diabetes, and have made it difficult for people with diabetes to control their blood sugar. Recent studies showed that more than half of the Saudi population consumed less than one serving of fruits and vegetables per day [40], and 96.1% of them were physically inactive [41]. Furthermore, 51.0% of the adult Saudi population were either overweight or obese [42] and the prevalence of overweight and obesity among people with diabetes was significantly higher than the general population in the country [43, 44]. In addition, studies from Saudi Arabia found an association between low physical activity and poor glycaemic control [10, 13, 35], while a study from Bahrain [45], a country that shares boundaries and similar culture with Saudi Arabia, showed an association between unhealthy eating habits and higher HbA1c among people with T2DM.

Healthy eating habits and regular physical activity are key components in the management of T2DM. The current guidelines for people with diabetes recommend 8–10 servings of fruits and vegetables every day [4]. A serving of fruits is equal to a medium-size apple, orange, or banana, while a serving of vegetables is half a cup of corn, carrot or leafy vegetables. The guidelines also recommend at least 150 min of moderate to vigorous intensity physical activity per week [4]. The majority (77.5%) of participants in this study, however, did not eat fruits and vegetables daily, which has increased their risk of inadequate and very poor glycaemic control. Similarly, more than two-thirds of participants (70.5%) did not achieve the recommended length of time of physical activity per week even though walking for transportation and physical activities at work were included in measuring it. Results of this current study also showed that a low level of physical activity was an independent risk factor for inadequate and very poor glycaemic control. Continuous education programs emphasising the role of lifestyle modification in controlling blood glucose level will be of great benefit for people with T2DM in Saudi Arabia.

Previous studies have shown that the more knowledge of diabetes a person has the more likely that he or she will have lower HbA1c level [17, 46]. Though the participants’ knowledge of the disease was not evaluated in this study, their awareness of HbA1c and its recommended level for people with diabetes was assessed as a proxy for knowledge of the disease. Only 31.9% of participants were aware of HbA1c and knew the recommended target (< 53 mmol/mol (< 7%)). The remaining participants either have not heard of HbA1c before (32.0%) or did not know the recommended HbA1c target (36.1%), which was associated with increased risk of inadequate and very poor control after adjustment for other risk factors. This finding is supported by the results of randomised control trials which showed that knowledge of actual and target HbA1c was associated with a significant reduction in HbA1c levels [47, 48]. In order to improve glycaemic control, physicians and health educators should ensure that people with diabetes are fully aware of their actual as well as the target HbA1c they should achieve.

Similar to studies conducted in the Arabian Gulf [49, 50] and other countries [18, 51] we found that younger age groups (≤60 years) were at higher risk of inadequate glycaemic control. Younger people are more likely to be affected by the change in lifestyle and less likely to be adherent to a management plan because of active occupational and social life [52]. Old people, in contrast, are less likely to be affected by the change in lifestyle and more likely to adhere to a management plan because they might be more concerned about their health, especially when they start to have comorbidities and complications [49]. Because of the beneficial effect of optimal glycaemic control on delaying complications, improving quality of life, and extending life expectancy among young people with diabetes [53], the management should aim at tight control once the diagnosis is made.

Another concerning finding of this study is that while the mean age of participants was 57.6 (±11.1) years (median: 57.8, 25th percentile: 51.8, 75th percentile: 63.9 years), they have a relatively long mean duration of diabetes of 13.9 (±8.4) years (median: 13.0, 25th percentile: 6.0, 75th percentile: 20 years). This indicates that the majority of people acquired diabetes in their early 40s. Early onset T2DM is associated with poor glycaemic control and a higher risk of comorbidities and complications [54]. With longer duration of T2DM, on the other hand, there is usually further deterioration of the function of the pancreas and the body’s resistance to insulin increases, which makes it more difficult to control blood glucose level. The likelihood of acquiring diabetes complications also increases with longer duration, and complications can negatively affect the control either directly through inflammation and disturbance of the body’s metabolism and indirectly through the effect of poly-pharmacy, anxiety, depression and stress. To prevent or delay diabetes and its complications, the healthcare system in Saudi Arabia should fully activate the screening programs and establish an intensive management protocols to identify and treat people at risk of diabetes.

Though BMI did not appear to affect glycaemic control, we found that a high waist-hip ratio was an independent risk factor for inadequate and very poor control. Similar findings were also observed in studies from Japan and the United States [55, 56]. In addition, compared to BMI, a high waist-hip ratio was found to have stronger association with cardiovascular disease among people with type 2 diabetes [57]. Waist-hip ratio is a more accurate measure of central obesity which is strongly linked with T2DM, poor glycaemic control, and cardiovascular disease [58]. Therefore, similar to BMI, waist-hip ratio should be measured and recorded for people with diabetes with every follow up visit, and health care providers should raise the awareness of people with diabetes about high waist-hip ratio and the risk associated with it.

We found that living in a remote village was a strong predictor of inadequate and very poor glycaemic control. People living in remote villages are likely to have a low level of formal education and are likely to have less access to fresh healthy food options. Because of accessibility issues, they limit their follow-up to the local primary health care centre and may not visit a specialised diabetes clinic or centre until the disease has progressed and they acquire complications. To improve the control among this susceptible group, the healthcare system should provide special diabetes education programs for healthcare providers working at remote places, and motivate them to use the online continuous education programs that are currently available and accredited by the Saudi Health System. General physicians working on remote sittings should also be provided with timely hotline or email access to specialists including endocrinologists, ophthalmologist and podiatrist. Patients, on the other hand, should have frequent teleconference or phone calls by diabetes educators, dietitians and other allied health professionals if these healthcare professionals are not assigned to the remote place where patients live.

Similar to other previous studies [18, 49, 50], our results showed that the use of injectable medications was a strong predictor for inadequate control. It is of concern that even after management with insulin, which is highly effective modality of treatment [59], a large proportion of people continued to have high blood glucose level. Low adherence to injectable medication regimen because of social stigmata, interference with daily activity, and fear of hypoglycaemia, have been suggested to increase the risk of poor control among people using injectable medications [60]. In Addition, the progression of the disease, weight gain related to insulin use, and polypharmacy can also contribute to poor glycaemic control among people with T2DM who are on injectable medications [61].

Our findings support the previous study that showed an association between inadequate glycaemic control and low income as well as low adherence to medications [18, 52, 62]. Low income decreases the likelihood of adherence to lifestyle modifications and treatment regimen [62], and low adherence to management plan is a known risk factors for poor glycaemic control [18, 52].

The association between hypoglycaemia and glycaemic control is of interest. Our findings showed that people with infrequent symptoms of hypoglycaemia were at higher risk of poor glycaemic control compared to those who had frequent symptoms of hypoglycaemic. This may indicate that while an intensive treatment regimen improve glycaemic control, it may come at the cost of frequent hypoglycaemia symptoms. Severe hypoglycaemia is associated with lower productivity, reduced quality of life, and a higher risk of anxiety, depression and mortality [63, 64]. Therefore, hypoglycaemia should be prevented, and the treatment should aim at achieving the lowest HbA1c level without severe hypoglycaemia episodes and a minimum number of mild hypoglycaemia symptoms [65].

The strength of this study lays on the relatively large sample size that was recruited from multiple centres from different regions of Saudi Arabia. The consideration of several potential risk factors and the use of a validated electronic questionnaire, which reduce data errors, also add strength to this study. This study, however, has some limitations. Cross-sectional study design lack temporality and causality cannot be inferred. Another limitation is that information regarding individualised glycaemic control targets could not be collected because it was not documented in participants’ medical records. Therefore, a HbA1c cut off point of 7% was selected to categorise adequate control which is too strict for old people with longer duration of the disease and those who have advanced cardiovascular disease [4]. In addition, we could not investigate the effect of new anti-hyperglycaemic agents such as the glucagon-like peptide-1 (GLP-1) receptor agonists on glycaemic control because only a very small number of participants in our database were using them. Nonetheless, this study clearly revealed the burden of inadequate glycaemic control among people with T2DM in Saudi Arabia and its associated risk factors.

## Conclusion

Inadequate glycaemic control is a common and widespread problem among people with T2DM in Saudi Arabia. Healthcare providers should undertake a patient-centred approach and individualise management strategies with consideration to all identified risk factors for inadequate control. Continuous education programs should also be implemented to raise the awareness of the disease and the importance of lifestyle modification. The healthcare system should prioritise diabetes prevention strategies through active screening and intensive management of people at risk. The health system should also take special measures to improve glycaemic control for people with diabetes living at remote locations. Future research should investigate the effectiveness of education programs targeting people with diabetes and barriers to adhering to lifestyle modifications.

## Abbreviations

BMI: Body Mass Index
eGFR: Estimated glomerular filtration rate
HbA1c: Glycated haemoglobin A1c
HDL: High density lipoprotein
LDL: Low density lipoprotein
T2DM: Type 2 diabetes mellitus
